# Efficacy and safety of simultaneous integrated boost intensity-modulated radiotherapy combined with temozolomide for the postoperative chemotherapy treatment of multifocal high-grade glioma

**DOI:** 10.3389/fonc.2025.1539362

**Published:** 2025-03-24

**Authors:** Nan Jiang, Li-ping Xu, Fei Li, Pei-pei Wang, Yuandong Cao

**Affiliations:** Department of Radiation Oncology, The First Affiliated Hospital with Nanjing Medical University, Nanjing, China

**Keywords:** multifocal high-grade glioma, simultaneous integrated boost intensity modulated radiotherapy, temozolomide, efficacy, toxicity

## Abstract

**Background:**

The multifocal manifestation of high-grade glioma is a rare disease with an unfavorable prognosis. The pathogenesis of multifocal gliomas and pathophysiological differences in unifocal gliomas are not fully understood. The optimal treatment for patients with multifocal high-grade glioma is not defined in the current guidelines; therefore, individual case series may be helpful as guidance for clinical decision-making.

**Methods:**

Patients with multifocal high-grade glioma treated with simultaneous integrated boost intensity-modulated radiotherapy combined with temozolomide for postoperative treatment at our institution between January 2020 and December 2023 were retrospectively analyzed. Multifocality was neuroradiologically assessed and defined as at least two independent contrast-enhancing foci in the MRI T1 contrast-enhanced sequence. Overall and progression-free survival were calculated from the diagnosis until death and from the start of radiation therapy until the diagnosis of disease progression on MRI for all patients.

**Results:**

A total of 42 patients with multifocal high-grade glioma were examined, of which 16 were female and 26 were male. The median age of all patients was 57 years (range: 23–77 years). The median KPS score was 80 (range: 50–100). Complete resection was performed in 10 cases, and partial resection was performed in 32 cases before the start of radiation therapy. The prescription schedule was 54 Gy (1.8 Gy × 30) with an SIB of 60 Gy (2 Gy × 30). Concomitant temozolomide chemotherapy was administered to 40 patients. Median survival was 19 months (95% CI 14.1–23.8 months) and median progression free survival after initiation of RT 13 months (95% CI 9.2–16.7 months). Five patients experienced grade 3 toxicity, none experienced grade 4 toxicity, and no treatment-related deaths occurred.

**Conclusion:**

Multifocal high-grade gliomas can be treated safely and efficiently with simultaneous integrated boost intensity-modulated radiotherapy with concomitant and adjuvant TMZ chemotherapy.

## 1 Introduction

The multifocal manifestation of high-grade glioma is less severe and has the most unfavorable prognosis. The median overall survival time is still reported to be as low as eight months in median, despite aggressive treatment (1). While the current World Health Organization (WHO) classification does not refer to multifocal high-grade glioma as a specific subentity (2), recent molecular studies have highlighted distinct genetic and epigenetic features that distinguish multifocal gliomas from their unifocal counterparts. Key molecular alterations include IDH mutations, MGMT promoter methylation, TERT promoter mutations, and alterations in the 1p/19q chromosomal status (3–6). These genetic markers not only influence the biological behavior of tumors but also impact their response to therapy. For instance, IDH mutations are associated with a relatively better prognosis, whereas MGMT methylation status is predictive of the response to temozolomide-based chemotherapy (4, 5). Understanding these molecular differences is crucial for developing targeted therapies and improving the outcomes of patients with multifocal high-grade gliomas.

In recent years, several studies have investigated clinical outcomes and treatment strategies for multifocal high-grade gliomas. Notably, a study by Haque et al. (1) compared the management patterns and outcomes of unifocal and multifocal glioblastomas, highlighting the challenges associated with multifocality. Another study by Paulsson et al. (6) compared the clinical outcomes and genomic characteristics of single-focus and multifocal glioblastomas, demonstrating the distinct molecular landscape of multifocal tumors. Additionally, Lahmi et al. (17) reported the use of whole-brain radiotherapy with concurrent temozolomide in newly diagnosed multifocal glioblastoma, achieving a median overall survival of 10 months. These studies collectively underscore the need for tailored treatment approaches and further research to improve outcomes in patients with multifocal high-grade gliomas. The current standard of care for newly diagnosed high-grade gliomas is maximal safe resection followed by radiotherapy (RT) in association with concomitant and adjuvant temozolomide (TMZ). Practice-changing studies on the treatment of high-grade glioma have included patients with multifocal tumors but did not analyze the prognosis and therapeutic outcomes of this subset of patients in detail (7–9). In current guidelines, the therapeutic management of patients with multifocal high-grade glioma is not defined separately from the treatment of unifocal high-grade gliomas (10–12). Recommendations on the best treatment for patients with multifocal high-grade glioma are still limited to institutional case series and database analyses.

Case series and database analyses of radiation therapy (RT) treatment of multifocal high-grade glioma patients have focused on different fractionation regimes compared to conventional fractionation with hypofractionated radiotherapy, as well as on the use of concomitant chemotherapy, leading to differing recommendations (1, 13–17). Unfortunately, multifocal high-grade glioma has been defined differently in many retrospective case series, and a multitude of different treatment regimens with only limited information about related adverse events have been reported, which limits the comparability of these analyses. Particularly, older case series without high-resolution MRI and state-of-the-art RT treatment techniques have limited transferability to the current treatment of patients with multifocal high-grade gliomas.

At our center, we used moderate SIB-IMRT combined with concomitant and adjuvant TMZ in postoperative patients with multifocal high-grade gliomas. The current study aimed to report the efficacy of this regimen, including treatment-related toxicity, local recurrence, progression-free survival (PFS), and overall survival (OS).

## 2 Methods

### 2.1 Patients

We retrospectively analyzed patients with a primary diagnosis of multifocal high-grade glioma who underwent RT at our department between January 2020 and December 2023. Patients who had histologically confirmed high-grade gliomas (2016 World Health Organization [WHO] grades III–IV) were included in this study. Patients underwent surgical resection before RT at our institution. Therefore, this study included only postoperative patients. No limitations were placed on the Karnofsky performance status (KPS), age, lesion location, or extent of surgery. This was a retrospective analysis, and approval was obtained from the institutional review board and the ethics committee.

### 2.2 Magnetic resonance imaging and definition of multifocal gliomas

MRI with contrast-enhanced T1 and T2 or FLAIR sequences was performed prior to RT for all patients. Only patients with multifocal growth patterns at the time of the first diagnosis, as assessed by an experienced neuroradiologist, were included in the study. High-grade gliomas were defined as multifocal gliomas, comprising at least two independent contrast-enhancing foci in the MRI T1 contrast-enhanced sequence.

### 2.3 Radiotherapy protocols

The indication for RT was based on the consensus recommendation of the interdisciplinary neuro-oncology tumor board for all cases. All patients were treated with limited-field irradiation. Prior to radiotherapy, a thermoplastic mask was individually made for each patient to ensure reproducibility of patient positioning during planning CT and the subsequent course of irradiation. The planning CT scan was performed at a slice thickness of 3 mm.

Radiation treatment plans included intensity-modulated radiation therapy (IMRT) and volumetric-modulated arc therapy (VMAT) plans. Irradiation regimens were administered as follows: 54 Gy (1.8 Gy × 30 Gy) with an SIB of 60 Gy (2 Gy × 30 Gy). Contrast-enhanced T1 sequences, T2, and/or FLAIR MRI sequences were co-registered with the planning CT images within the Monaco treatment planning system (version 5.11, Elekta, Sweden).

Concomitant and adjuvant chemotherapy was administered according to the protocol of the EORTC 26,981/22981 NCIC CE.3 trial (7), with temozolomide 75 mg/^2^every day during RT. After a 4-week break, the patients received adjuvant TMZ (150–200 mg/m^2^/day) for 5 days every 28 days. The total number of TMZ cycles was determined by oncologists according to patients’ general condition, compliance, economic situation, and disease progression.

### 2.4 Target volumes

Gross tumor volume (GTV) was defined as all contrast-enhancing lesions on postoperative MRI T1-weighted images and the postoperative cavity with the latter fused with computed tomography images for treatment planning. The clinical target volume (CTV1) was defined as the GTV plus a 1-cm margin, including surrounding edema on T2-weighted fluid-attenuated inversion recovery MRI. CTV2 was defined as the GTV plus a 2-cm margin. The planning target volume (PTV), including PTV1 and PTV2, was defined as the respective above target volume plus a 0.3-cm margin. CTV1 received 60 Gy and CTV2 received 54 Gy. The margin could be modified to a smaller margin if there are organs at risk (OARs), such as the brain stem, optic pathways, or spinal cord, or if there are anatomical barriers, such as the dura, tentorium, and falx cerebri.

### 2.5 Statistical and survival analysis

Statistical analyses were performed using IBM SPSS Statistics version 25 (IBM, Armonk, NY, USA). The Kaplan–Meier method was used to evaluate the rates of local recurrence, PFS, and OS. Overall survival was calculated as the time interval between histological confirmation and the date of death or loss to follow-up. Progression-free survival was assessed as the time interval between the initiation of RT and the first imaging detection of progressive disease according to the RANO criteria (18) or loss to follow-up.

### 2.6 Follow-up

Patients were followed-up weekly during the treatment period, with a medical history, physical examination, and complete blood test, and were followed-up every month after finishing the treatment for at least 3 months. All patients underwent routine neurological examinations and MRI at 3-month intervals after treatment. MR spectroscopy and perfusion of the brain were not routinely used, except when there was doubt about tumor progression or necrosis. Acute toxicities were scored using the Common Terminology Criteria for Adverse Events, version 4.0. Late toxicities were scored according to RTOG/EORTC toxicity criteria.

## 3 Results

### 3.1 Patients

Between January 2020 and December 2023, 42 patients with multifocal high-grade glioma were examined, of which 16 were female and 26 were male. The median age of all patients was 57 years (range: 23–77 years). The median KPS score was 80 (range: 50–100). The patient characteristics are shown in **Table 1**.

**Table 1:** Demographic and baseline clinical characteristics of patients.

Characteristic		Patients(No, %)
Sex	male	26 (61.9%)
	female	16 (38.1%)
Age(year)	Median (range)	57 (23–77)
	≤50	10 (23.8%)
	>50	32 (76.2%)
Extent of surgery	Partial resection	32 (76.2%)
	Complete resection	10 (23.8%)
KPS scores	90–100	9 (21.4%)
	80	20 (47.6%)
	≤70	13 (31.0%)
TMZ cycles	Median (range)	6 (0–12)
	<6	15 (35.7%)
	≥6	27 (64.3%)
Principal Symptoms	Headache	15 (35.7%)
	Seizures	10 (23.8%)
	Motor Weakness	8 (19.0%)
	Cognitive Decline	5 (11.9%)
Histological Diagnosis with WHO Grade	Glioblastoma (WHO IV)	30 (71.4%)
	Anaplastic Astrocytoma (WHO III)	8 (19.0%)
	Diffuse Midline Glioma (WHO IV)	4 (9.5%)
Molecular Characteristics		
IDH Mutation Status	IDH-wildtype	38 (90.5%)
	IDH-mutant	4 (9.5%)
MGMT Methylation Status		
	MGMT-methylated	18 (42.9%)
	MGMT-unmethylated	24 (57.1%)
Radiotherapy Volumes	Conformity Index	0.85 ± 0.05
	Homogeneity Index	0.90 ± 0.03
	Median Dose to Hippocampus (Gy)	12.0 ± 1.5

### 3.2 Efficacy

**Figure 1:**
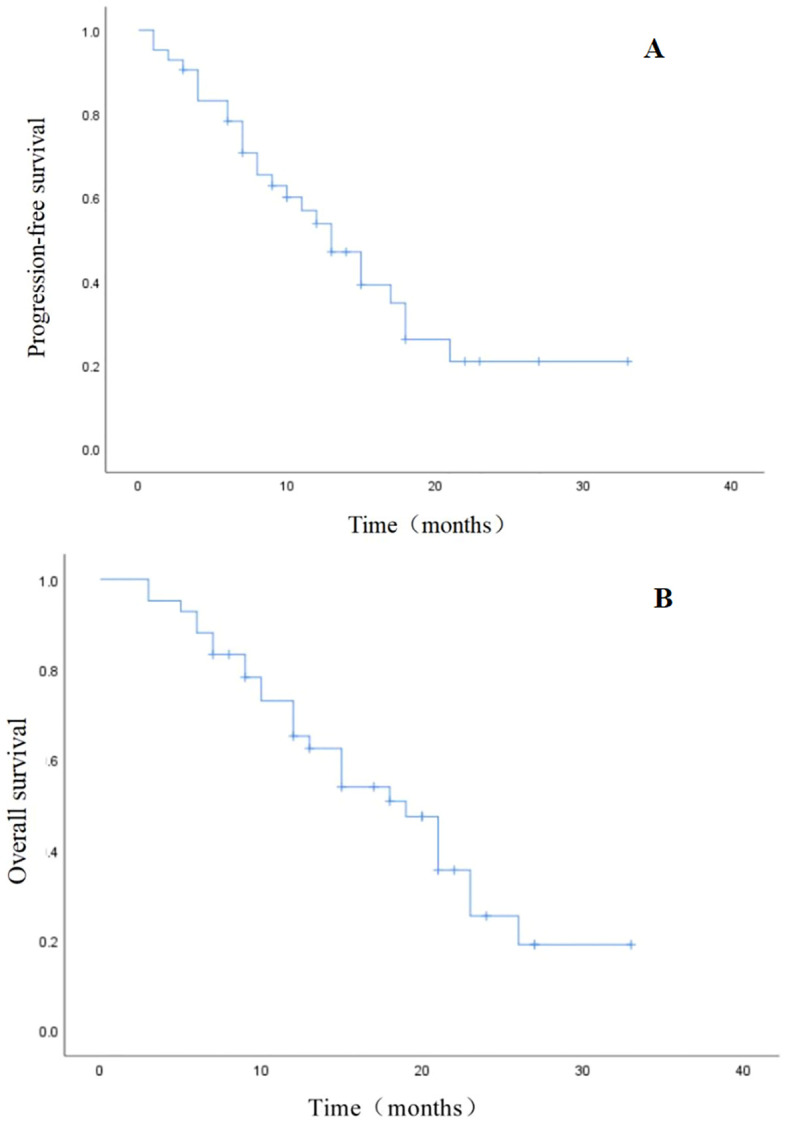
Kaplan–Meier estimates. **(A)** Progression-free survival (PFS). **(B)** Overall survival (OS).

After a median follow-up of 15 months (range: 3–33 months), 26 (61.9%) patients died, and 31 (73.8%) patients exhibited tumor progression. The median OS and PFS rates were 19 (95% confidence interval [CI], 14.1–23.8) and 13 (95% CI, 9.2–16.7), respectively. The 1- and 2-year PFS rates among the whole group were 57.1% and 38.1%, respectively (**Figure 1A**). The 1- and 2-year OS rates were 66.7% and 40.5%, respectively (**Figure 1B**).

### 3.3 Patterns of failure

Tumor progression was detected in 31 patients (73.8%), and progression was identified on MRI (**Table 2**). Ten patients had tumor progression within the GTV, three patients developed new lesions within CTV1, two patients developed new lesions within CTV2, six patients developed new lesions outside the radiation field, and three patients developed multicentric recurrence. The progression sites of the seven patients were unknown because MRI findings were unavailable. The remaining nine patients were alive with no evidence of progression.

**Table 2:** Patterns of recurrence.

Sites of recurrence	Number of patients (N = 31)
Within GTV	10
Within CTV1	3
Within CTV2	2
Outside the target volume	6
Multicentric recurrence	3
Unknown	7

### 3.4 Treatment

Concomitant and adjuvant chemotherapy was administered according to the protocol of the EORTC 26,981/22981 NCIC CE.3 trial (7), with temozolomide 75 mg/^2^every day during RT. After a 4-week break, the patients received adjuvant TMZ (150–200 mg/m^2^/day) for 5 days every 28 days. The total number of TMZ cycles was determined by oncologists according to patients’ general condition, compliance, economic situation, and disease progression. Treatment at progression was best supportive care in 16 cases, combined bevacizumab in eight patients and TTField treatment in two cases. In nine cases, there was no progression at the time of data analysis.

### 3.5 Adverse events

The most common acute toxicities were nausea, fatigue, headache, and hematologic toxicities, which were mainly grade 1 or 2 and occurred during the concomitant RT and TMZ periods. Five patients experienced grade 3 toxicities, including neutropenia (one patient, 1.3%), anemia (two patients, 2.5%), and thrombocytopenia (two patients, 2.5%). None of the patients experienced grade 4 toxicities. The most common late adverse effects were cognitive disturbances, which occurred in four patients (5.0%). Three patients (3.7%) developed radionecrosis. Two patients (2.5%) presented with progressive headache and dizziness 1 year after RT, and MRI showed increased enhancement. The patients underwent reoperation. Postoperative pathological examination revealed only necrotic tissue and no tumor tissue. One patient (1.2%) was symptom-free, but MRI showed increased enhancement 3 months after treatment. However, there was a lack of evidence of progression upon MR perfusion and MR spectroscopy; therefore, a diagnosis of clinical radionecrosis was made. All the patients completed the planned RT treatment. No treatment-related deaths occurred in this study (**Table 3**).

**Table 3:** Adverse events.

Adverse events	Grade 1 toxicities	Grade 2 toxicities	Grade 3 toxicities	Grade 4 toxicities
hematologic toxicities				
neutropenia	2	–	1	–
anemia	4	1	2	–
Thrombocytopenia	1	2	3	–
ALT and AST increase	3	2	–	–
Fatigue	5	3	–	–
Headache	2	1	–	–
Alopecia	30	12	–	–
Nausea	9	6	–	–
Dermatitis	2	-	–	–
Dizziness	10	6	–	–
Cognitive disturbance	2	2	–	–

## 4 Discussion

There are currently no definitive standards for the diagnosis and treatment of patients with multifocal high-grade gliomas. The current WHO classification of tumors of the central nervous system does not differentiate between multifocal and unifocal high-grade gliomas (2), although several studies on the histopathology of multifocal high-grade gliomas have postulated that distinct histopathological differences are observed (3–6). Current guidelines do not address multifocal high-grade glioma separately from the unifocal presentation of the disease (10). Since few investigator-initiated trials on RT treatment exist (19), it is not surprising that there is very limited evidence for the radiotherapeutic treatment of patients with multifocal glioblastoma.

To better understanding of the multifocal form of high-grade glioma, we examined a set of 42 unselected multifocal high-grade glioma cases treated with conventionally fractionated, limited-field RT with modern techniques including IMRT and VMAT with concomitant chemotherapy. The focus of this case series was the assessment of treatment outcomes in terms of progression-free survival, and treatment-related adverse events. Our study demonstrated the efficacy and safety of simultaneous integrated boost intensity-modulated radiotherapy combined with temozolomide for the postoperative treatment of multifocal high-grade glioma. These findings highlight the potential benefits of our treatment approach in our patient cohort. However, further prospective studies are warranted to confirm these results and determine the optimal treatment strategy for this challenging disease (20–22).

Because of the aggressive treatment approach, progression-free and overall survival in the present cohort were markedly superior to other high-grade glioma cohorts with predominantly unifocal tumors treated with RT and concomitant daily temozolomide, with a median overall survival of up to 15.7 months (7, 8, 23). In previous RT case series of multifocal high-grade glioma patients treated with modern treatment techniques, the overall survival was comparable to our case series, with reported median overall survival times in the range between 8.2 months (6), 8.7 months (13), and 11.5 months (16).

Although our survival outcome seems to be superior to the results reported by most other studies, it is difficult to compare our results with those of these studies directly because the definitions of target volumes and fractionation schedules employed in these studies vary widely. In addition, other studies not only included patients who had undergone surgical resection, but also those who received only biopsy, whereas our study included only patients who had undergone surgery. Finally, while other studies may only include WHO grade 4 gliomas, our study included a small number of grade 3 gliomas. Only a well-designed randomized trial can confirm whether our regimen is comparable to or superior to the standard treatment regimen.

One of the main reasons for the poor overall survival of multifocal high-grade glioma patients could be the reduced performance status of the patients, which was also evident in the present cohort with a median KPS of 80 prior to the initiation of RT and 80 at the end of RT. A KPS above the median prior to and at the end of RT, respectively, showed a trend towards longer survival in this series, even though statistical evaluations must be considered with caution due to the small number of cases.

Histopathologically, it has been discussed that the higher phenotypic aggressiveness of multifocal glioma itself might explain the poorest survival of all glioma subtypes (6, 24). The risk of refractory edema caused by large tumor infiltration and large RT treatment volumes, with the prolonged need for dexamethasone after the completion of RT, can also be discussed as a reason for poorer overall survival in patients with multifocal tumors.

Interestingly, the three cases with grade 3 edema were cases with PTV volumes below or within the range of the median; therefore, the PTV volume by itself may not be the determining factor for the occurrence of edema after radiotherapy.

Whole brain radiotherapy (WBRT), which is the standard of care prior to the introduction of 3D conformal RT, had considerably worse treatment outcomes, with reported median overall survival times of only 3.7 months (13). However, a recently reported monocentric case series of WBRT with concomitant and adjuvant TMZ chemotherapy in patients with newly diagnosed multifocal glioblastoma reported a median OS of 10 months. The reported toxicities of this WBRT series were comparable to the limited field RT of this series, with three grade 3 toxicities and one grade 4 toxicity (17).

A recent large-scale study initiating a nomogram for survival prediction in glioblastoma patients and a subsequent validation study showed that a low KPS and lack of gross total resection, as present in the current case series, are significantly correlated with poorer overall survival. Notably, multifocality itself was not included in this nomogram, possibly because of the rarity of this condition (25, 26). In contrast, radiomics approaches, which are increasingly used for prognostic assessment of glioblastoma patients, used multifocality as one of the main imaging features (27–29).

Large database studies have shown that concomitant systemic treatment with temozolomide has a benefit specifically in patients who cannot undergo surgical resection of the tumor, in both unifocal and multifocal growth patterns (1, 15). Nevertheless, further information about toxicities related to concomitant temozolomide in patients with multifocal high-grade glioma patients could not be determined in these studies, as it was not documented in the databases. In our series, concomitant chemotherapy with temozolomide was administered to 40 patients with mostly acceptable toxicity, only five patients developed grade 3 hematologic toxicities.

In our unselected limited-field RT cohort, adverse events were manageable despite the relatively larger irradiated brain volume. Only three cases developed radionecrosis. Two patients presented with progressive headache and dizziness 1 year after RT. Two patients presented with new-onset seizures possibly related to radiation treatment and increasing cerebral edema, which did not appear to be related to the size of the PTV volume or above the median values of V30, V45, and D2 of the brain.

Given the retrospective nature of our study and the inclusion of only operated patients, our findings may have been subject to selection bias. Specifically, the presence of WHO grade III tumors in our cohort, along with non-randomized treatment allocation, may limit the generalizability of our results. Although our treatment approach demonstrated promising efficacy and safety profiles, these findings should be interpreted with caution. Future prospective studies with larger and more diverse patient populations are needed to validate our results and to better understand the true impact of our treatment regimen.

## 5 Conclusion

In this case series, multifocal high-grade glioma was treated safely and efficiently with simultaneous integrated boost intensity-modulated radiotherapy with concomitant and adjuvant TMZ chemotherapy, and the survival outcome was better than that in other studies. Prospective studies are warranted to select the best treatment regimen for patients with multifocal high-grade glioma to improve the oncological outcomes.

## Data Availability

The raw data supporting the conclusions of this article will be made available by the authors, without undue reservation.
